# Microwave Resonant Probe-Based Defect Detection for Butt Fusion Joints in High-Density Polyethylene Pipes

**DOI:** 10.3390/polym17192617

**Published:** 2025-09-27

**Authors:** Jinping Pan, Chaoming Zhu, Lianjiang Tan

**Affiliations:** 1 School of Mechanical and Electrical Engineering, China University of Mining and Technology, Xuzhou 221116, China; 2 Jiaxing Special Equipment Inspection and Testing Institute, Jiaxing 314000, China; 3 School of Materials Science and Engineering, Shanghai Institute of Technology, Shanghai 201418, China

**Keywords:** non-destructive testing, microwave resonant probe, HDPE pipes, butt fusion joints, defect detection

## Abstract

With the widespread use of high-density polyethylene (HDPE) pipes in various industrial and municipal applications, ensuring the structural integrity of their joints is crucial. This paper presents a novel defect detection method based on a microwave resonant probe, designed to perform efficient and non-destructive evaluation of butt fusion joints in HDPE pipes. The experimental setup integrates a microwave antenna and resonant cavity to record real-time variations in resonance frequency and S_21_ magnitude while scanning the pipe surface. This method effectively detects common defects, including cracks, holes, and inclusions, within the butt fusion joints. The results show that the microwave resonant probe exhibits high sensitivity in detecting HDPE pipe defects. It can identify different sizes of cracks and holes, and can distinguish between talc powder and sand particles. This technique offers a promising solution for pipeline health monitoring, particularly for evaluating the quality of welded joints in non-metallic materials.

## 1. Introduction

High-Density Polyethylene (HDPE) pipes are widely recognized for their superior properties, including high strength-to-density ratio, corrosion resistance, chemical inertness, durability and flexibility [1,2]. These attributes make HDPE pipes an ideal solution for fluid and gas transportation in various industrial and municipal applications [3,4,5,6]. Compared to traditional materials like metal or concrete, HDPE pipes offer a longer service life and superior performance in corrosive environments.

The integrity and reliability of HDPE piping systems depend heavily on the joining methods employed. The most commonly used joining techniques are butt fusion welding and electrofusion welding, with butt fusion being even more reliable for large-diameter pipelines [7]. Ensuring the structural integrity of HDPE pipes is critical for system performance. Several non-destructive testing (NDT) and inspection techniques are applied [8], such as ultrasonic testing (UT), thermal imaging, acoustic emission, and microwave NDT, a newer technique that can evaluate process-related defects and monitor weld quality in non-metallic materials [9].

Microwave NDT operates on the principle of electromagnetic wave interaction with the material under test [10,11,12]. HDPE, being a dielectric material with low loss, allows microwaves to penetrate and interact with its internal structure. Variations in dielectric constant caused by defects, such as voids, inclusions, or incomplete fusion in weld joints, alter the reflected or transmitted signals, enabling defect detection and characterization [13]. Microwave NDT has emerged as a promising technique for evaluating the quality of HDPE pipes, particularly for detecting defects in joints, assessing material uniformity, and identifying structural anomalies [14,15]. For instance, microwave reflectometry has been successfully implemented on in-line inspection robots to identify cracks and other anomalies in nonmetallic pipelines [16]. Additionally, microwave imaging techniques have been benchmarked against phased array ultrasonic testing, showing comparable or superior results in detecting defects in HDPE pipes [17]. While these microwave NDT methods have demonstrated excellent detection performance, challenges persist, such as signal attenuation and the requirement for specialized equipment. More critically, existing research indicates that different types and locations of defects often necessitate the use of distinct microwave probes and frequencies for effective detection. This variability significantly complicates the integration of microwave NDT technologies into automated inspection systems, posing a barrier to their widespread adoption and operational efficiency. Additionally, detection of impurity inclusion in the butt fusion joints has been rarely investigated.

We used horn antennas to detect defects in HDPE pipes. However, satisfactory results were not obtained. To enable the detection of common defects and flaws in HDPE pipelines at a single frequency, we designed a probe based on the principle of microwave resonance. The probe consists of a microwave antenna and a resonant cavity, which is connected to a vector network analyzer (VNA) to form a complete microwave detection system. This system translates the impact of the tested sample on the antenna’s radiation into variations in the resonant parameters of the cavity, ensuring high sensitivity for defect detection. This study primarily focuses on the detection of cracks and holes on the outer surface of HDPE pipelines, as well as the identification of inclusions and contaminants within the butt fusion joints of HDPE pipes. The experimental results demonstrate that the proposed microwave detection system is highly effective in accurately identifying these defects and damages, showcasing its potential for practical applications in pipeline integrity assessment.

## 2. Experimental Methods

### 2.1. Materials

HDPE pipes (PE100, DN200) with a nominal diameter of 200 mm were provided by Haining Chinaust Plastic Piping System Co., Ltd. (Jiaxing, China). The HDPE pipes were connected by butt fusion at a temperature of 220 °C and under the pressure of 1.0 MPa. The relative permittivity *ε*_r_ of HDPE pipes is 2.58 ± 0.07, measured by VNA based on the transmission line method. The number of included talc powder and sand particles is 5.0 ± 0.03 and 3.8 ± 0.02, respectively.

### 2.2. Problem Statement

HDPE pipes are prone to various defects that may arise during manufacturing or installation. This study addresses this problem by applying microwave nondestructive testing (NDT) to HDPE pipes, aiming to evaluate its capability in detecting and differentiating between different defect types, including holes, cracks, and inclusions of talc powder sand particles.

### 2.3. Simulation

In order to obtain detailed information on the microwave resonant probe, the electromagnetic and antenna simulations for the probe, including the radiation pattern and the near-field electric field distribution, were performed using Ansys High-Frequency Structure Simulator (HFSS) software (v. 2023R1). The simulation was conducted over a frequency range of 23.00–23.20 GHz. The model geometry, which incorporates the resonant cavity and the focused antenna, was designed according to the physical configuration of the system. The dielectric properties of HDPE, including its relative permittivity (*ε*_r_ = 2.58), were defined according to material specifications. A fine mesh around the interaction region between the probe and the HDPE pipe was used, ensuring accurate resolution of the electromagnetic field distribution at the probe’s focal point. The minimum mesh size was 0.6–1.0 mm, and 10–15 mesh elements per wavelength were used. Radiation boundaries at the outer edges of the simulation domain was applied to represent the open space surrounding the probe. In addition, a perfect electric conductor boundary at the metallic parts of the probe were utilized to model ideal conductive materials.

### 2.4. Microwave Test Systems and Principles

**Figure 1:**
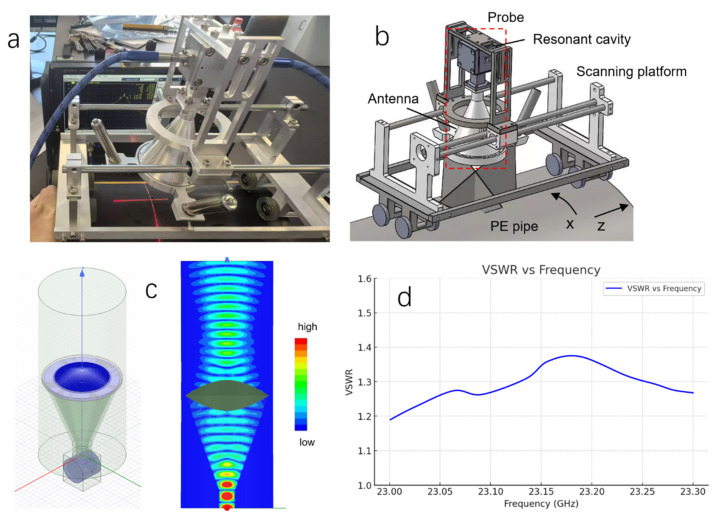
(**a**) The microwave-based testing system specifically designed for the inspection of HDPE pipelines. (**b**) Simulated microwave testing system showing the major components and the scanning directions. (**c**) Simulated characteristic diagrams of the resonant cavity-coupled focusing antenna: radiation pattern (**left**); near-field electric field distribution (**right**). (**d**) Plot of VSWR versus frequency curve for the resonant probe.

We have, for the first time, achieved the detection of defects in HDPE pipes using a microwave resonant probe, which is entirely different from the existing microwave testing methods. As shown in Figure 1a,b, the microwave resonant probe primarily consists of a focused antenna and a resonant cavity with two ports: a strongly coupled port and a weakly coupled port. The strongly coupled port is connected to the focused antenna via a circular-to-rectangular waveguide transition, while the weakly coupled port is linked to a Vector Network Analyzer (VNA, Keysight Technologies N5222B, Santa Rosa, CA, USA) through a microwave cable. The focused antenna is equipped with a conical horn and a double-convex dielectric lens, positioned at the horn’s aperture. The focal length can be adjusted by selecting different lenses as needed. This probe is specifically designed for operation within the K_a_, K, and K_u_ frequency bands.

Figure 1c shows the simulated electric field distribution within the resonant cavity and in the near field of the probe. The microwave energy is concentrated in the near field, ensuring that the microwaves are focused on the target location on the sample. During the HDPE pipe inspection, the microwave resonant probe is mounted on a scanning platform (Figure 1a,b) and placed against the outer surface of the pipe, with the standoff distance—i.e., the distance between the focused antenna and the pipe surface—set equal to the antenna’s focal length. The probe then performs a two-dimensional scan along both the circumferential (x-axis) and axial (z-axis) directions of the pipe (Figure 1b), while a laser positioner records the coordinates of the probe’s position.

In this study, butt fusion joints of HDPE pipes were tested at a microwave resonant frequency of 23.14 GHz, which is well-matched to the probe’s operating frequency and provides high sensitivity to HDPE materials. A double-convex lens with a focal length of 50 mm was chosen, resulting in a consistent standoff distance of 50 mm throughout the detection process. The VSWR near 23.14 GHz was measured, as shown in Figure 1d. VSWR quantifies the degree of reflection in the transmission line and indicates the efficiency of the probe in radiating microwave energy. The measured VSWR values, ranging from 1.19 to 1.37, suggest low reflection loss and high emission efficiency near the resonance frequency.

The microwave resonant probe is connected to Ports 1 and 2 of the VNA. During detection, the point-focused antenna concentrates the energy radiated by the resonant cavity at the focal point. When the radiated microwaves interact with the sample, a portion of the microwaves is reflected back. This reflected signal interacts with the electromagnetic field inside the resonant cavity, causing shifts in the cavity’s resonant parameters. The VNA records the real-time resonance curve, displaying the variation in the magnitude of the S-parameter S_21_ as a function of frequency within the resonance range. S-parameters are commonly used in microwave engineering to characterize the behavior of devices when subjected to incident electromagnetic waves. They describe how power is reflected or transmitted at different ports of the device under test. S_21_ represents the transmission coefficient, which measures the power passing from port 1 to port 2. In our detection system, the microwave signal is output from Port 1 of the VNA and transmitted through the microwave resonant probe toward the sample under test. The reflected signal is then received by the probe and returned to Port 2 of the VNA. Therefore, the S-parameter measured by this system is S_21_. Changes in the resonance curve, such as shifts in the resonance peak or frequency, reflect alterations in the resonant parameters due to the sample. These resonant parameters are highly sensitive to variations in the sample, enabling the detection of cold weld defects in the butt fusion joints of HDPE pipes.

## 3. Results and Discussion

**Figure 2:**
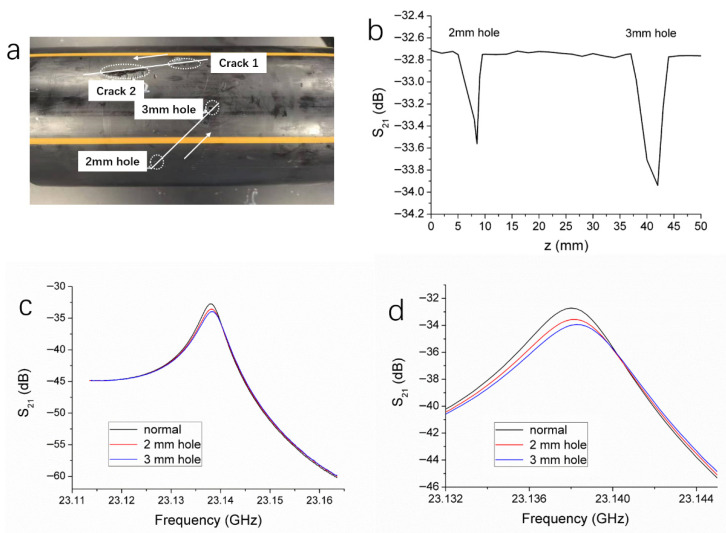
(**a**) A picture of an HDPE pipe with holes and cracks on the wall surface. (**b**) S_21_ magnitude versus scanning distance for the holes as the microwave probe scans along the white line. The white arrow indicates the scanning direction. (**c**) S_21_ versus frequency (resonance curves) obtained when the microwave probe is located at a defect-free position, the 2 mm hole, and the 3 mm hole on the pipe wall surface, respectively. (**d**) The enlarged view of the resonance curves near the resonance peaks in (**c**).

An HDPE pipe with defects of holes and cracks on the wall surface is shown in Figure 2a. There are two through-holes on the pipe wall with diameters of 2 mm and 3 mm, respectively; these holes penetrate the entire thickness of the pipe wall. Besides, there are two cracks of different sizes on the wall of the same pipe. Specifically, crack 1 has a length of 20 mm and a depth of 1.5 mm, while crack 2 measures 30 mm in length and 3 mm in depth. The white arrows in the figure indicate the scanning direction of the microwave probe.

Since the holes on the pipe wall are through-holes, they all share the same depth (equal to the pipe wall thickness). Therefore, this study focuses solely on the detection of holes with different diameters. During the microwave resonant probe’s inspection of the HDPE pipe, the probe is maintained at a constant distance from the pipe wall and scans along the direction indicated by the arrow in Figure 2a, with a step size of 1 mm. This step size was chosen as a compromise between spatial resolution and experimental conditions. As the probe passes over holes with diameters of 2 mm and 3 mm, the VNA records the variation in the S_21_ magnitude of resonance peaks with respect to the scanning distance. The results are shown in Figure 2b. It is observed that when the probe scans areas without defects, the S_21_ curve remains relatively stable with minimal fluctuation, indicating that the microwave signal reflected back into the resonant cavity is steady and that the resonant state is not significantly affected. When the probe moves to the location of the 2 mm hole, a pronounced dip appears in the S_21_ curve, signifying a substantial decrease in the S_21_ magnitude. After passing over the defect, the curve returns to its stable state. A second dip occurs when the probe is against the 3 mm hole. This dip is deeper than the one caused by the 2 mm hole, indicating a greater reduction in the S_21_ magnitude. Furthermore, the width of the dip corresponding to the 3 mm hole is broader than that of the 2 mm hole.

Each point on the S_21_ magnitude versus scanning distance curve corresponds to a specific resonance curve. By recording the resonant peak S_21_ magnitude at each point during the scanning process, a complete scanning curve is obtained. Figure 2c illustrates the resonance curves at the minimum points of the dips corresponding to the 2 mm and 3 mm holes, as well as at a defect-free location. Figure 2d presents a magnified view of the resonant peak regions from the three resonance curves shown in Figure 2c. All three resonance curves fall within the frequency range of 23.135 to 23.140 GHz. As shown in Figure 2d, compared to the defect-free location, the resonance peak magnitude at the 2 mm hole decreases by 0.85 dB. For the 3 mm hole, the resonance peak magnitude decreases by an additional 0.63 dB, and the peak also exhibits a slight frequency shift.

**Figure 3:**
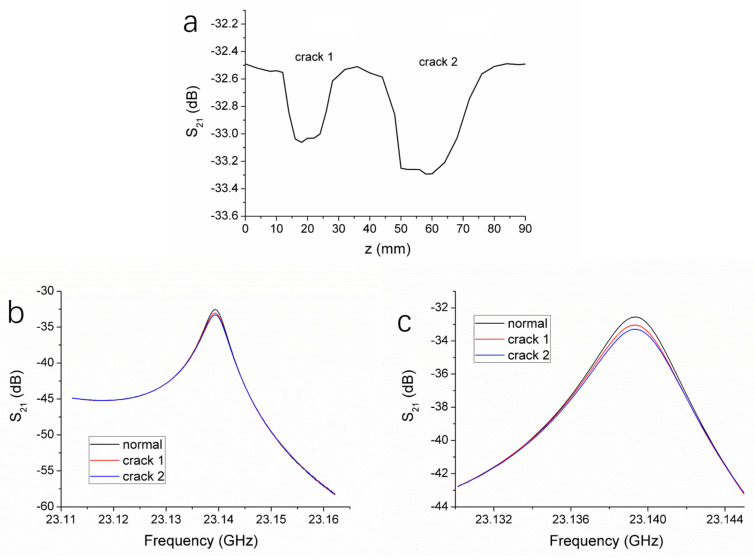
(**a**) S_21_ magnitude versus scanning distance for the cracks as the microwave probe scans along the direction indicated by the white arrow in Figure 2a. (**b**) Resonance curves obtained when the microwave probe is located at a defect-free position, the crack 1, and the crack 2 on the pipe wall surface, respectively. (**c**) The enlarged view of the resonance curves near the resonance peaks in (**b**).

The surface crack defects on the HDPE pipe wall were examined using the same detection method as for the hole defects. The probe scanned in the direction indicated by the arrow in Figure 2a, with a step size of 1 mm. As the probe passed over the crack 1 (30 mm in length, 3 mm in depth) and the crack 2 (20 mm in length, 1.5 mm in depth), the variation in the resonance peak magnitude with respect to the scanning distance was recorded, as shown in Figure 3a. It can be observed that when the probe scans regions without defects, the S_21_ curve remains relatively stable, with minimal fluctuations. This indicates that the microwave signal reflected back into the resonant cavity is steady and that the resonant condition remains largely unchanged. When the probe moves to the edge of crack 2, the S_21_ curve begins to trend downward. As the probe continues moving, a pronounced dip gradually forms, with the minimum S_21_ magnitude at the bottom of the dip reaching −33.35 dB. Once the probe has moved past the crack, the S_21_ curve returns to a stable state. A similar dip appears when the probe reaches the location of crack 1, with the minimum S_21_ magnitude being −33.02 dB. Analysis of the scanning curve reveals that deeper cracks result in more pronounced dips in the S_21_ magnitude. Furthermore, the width of the dip is observed to be proportional to the length of the crack, making it possible to approximately estimate the crack length based on the distance over which the S_21_ magnitude decreases and then recovers.

The resonance curves corresponding to the minimum points of the two dips, as well as that of a defect-free region, are shown in Figure 3b. Figure 3c provides a magnified view of the resonance peak regions for the three curves. All three resonance curves lie within the frequency range of 23.135 to 23.140 GHz. As shown in Figure 3c, the resonance peak magnitudes drop significantly when the probe passes over the cracks, compared to the defect-free region. The extent of this magnitude reduction is primarily influenced by the depth of the crack, while the length of the crack mainly affects the width of the dip in the scanning curve. It is also evident from Figure 3c that the presence of cracks has minimal impact on the resonant frequency.

**Figure 4:**
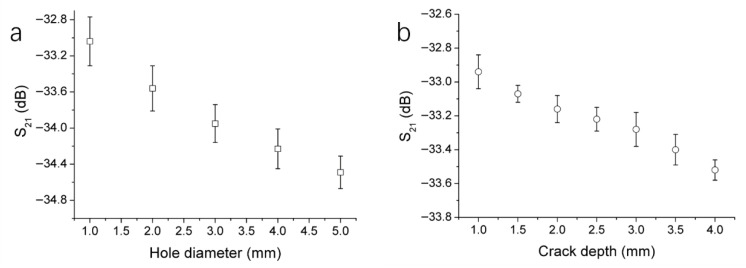
Changes of S_21_ magnitude at the resonance peak with the hole diameter (**a**) and the crack depth (**b**). The error bars represent the standard deviation (n = 4).

To further investigate the microwave nondestructive testing system’s ability to distinguish hole sizes and crack depths, additional holes of different diameters and cracks of various depths were examined. In addition to the 2 mm and 3 mm holes, holes with diameters of 1 mm, 4 mm, and 5 mm were also tested, using the same method as for the 2 mm and 3 mm holes to obtain their S_21_ magnitudes at resonance peaks. Each of the five different holes was tested four times. The average S_21_ magnitudes obtained were plotted against hole diameter, as shown in Figure 4a. It can be observed that as the hole diameter increases, the S_21_ magnitude decreases. Therefore, the size of the hole defect can be determined by the degree of reduction in the S_21_ magnitude. In comparison to the effect on the S_21_ magnitude, the hole diameter has little impact on the resonance frequency, especially when the hole diameter increases to 3 mm or more, at which point the resonance frequency no longer increases significantly with the hole size. Hence, changes in the S_21_ magnitude serve as the primary criterion for detecting hole defects.

We also conducted tests on five additional cracks with varying depths, specifically 1 mm, 2 mm, 2.5 mm, 3.5 mm, and 4 mm. Each of the seven cracks was tested four times, and the average S_21_ magnitudes obtained were plotted against crack depth, as shown in Figure 4b. It can be seen that as the crack depth increases, the S_21_ magnitude decreases. Thus, the depth of the crack can be inferred from the degree of reduction in the S_21_ magnitude. The depth and length of the crack have ignorable impact on the resonance frequency. Therefore, by combining changes in the S_21_ magnitude and the width of the dip in the scanning curve, crack defects can be effectively detected.

The system operates in the propagating wave regime, with the standoff distance placing the measurement within the transition region between near-field and far-field. This strikes a balance between the high spatial resolution characteristic of the near-field and the signal stability of the far-field. When the probe moves along the HDPE pipe wall, the reflected signal remains relatively stable in defect-free regions, and the resonant state of the microwave within the resonant cavity remains unchanged. As a result, the S_21_ magnitude of the resonance peak remains nearly constant. When the probe passes over a hole defect, the microwave reflection changes because the relative dielectric constant of air inside the hole is 1, which is significantly different from that of HDPE. When microwaves propagate to the interface between two media with different dielectric constants, the propagation characteristics change, and the reflection increases. HDPE is a non-magnetic polymer with relative permeability *μ*_r_ ≈ 1, identical to free space. Its *μ*_r_ remains nearly constant under electromagnetic fields. Changes in permeability can thus be ignored during microwave testing. The following formula can be used to describe the reflection of microwaves at the interface [18]:

(1) $$R=\frac{\sqrt{\varepsilon_{2}}-\sqrt{\varepsilon_{1}}}{\sqrt{\varepsilon_{2}}+\sqrt{\varepsilon_{1}}}$$

where *R* represents the reflection coefficient, indicating the strength of microwave reflection at the interface of the media, with a value range of [−1, 1]. The greater the absolute value of *R*, the stronger the reflected microwave energy and the greater the reflection loss. *ε*_1_ and *ε*_2_ represent the relative dielectric constants of the two media or materials. When microwaves propagate from medium 1 to medium 2, part of the microwave energy is reflected back, while the rest is transmitted into medium 2. The magnitude of *R* reflects the strength of the reflected signal. On the other hand, the sign of *R* indicates the phase inversion of the reflected wave relative to the incident wave. If the reflection coefficient is positive, the reflected wave has the same phase as the incident wave; if the reflection coefficient is negative, the reflected wave has the opposite phase of the incident wave. Microwaves reflect at the interface between air and HDPE, and the reflected wave enters the resonant cavity. The reflected wave, which is out of phase with the incident wave, causes destructive interference with the microwaves within the resonant cavity, resulting in a decrease in the S_21_ magnitude. The reduction in S_21_ magnitude suggests that the reflected wave, which is out of phase with the incident wave, predominates.

**Figure 5:**
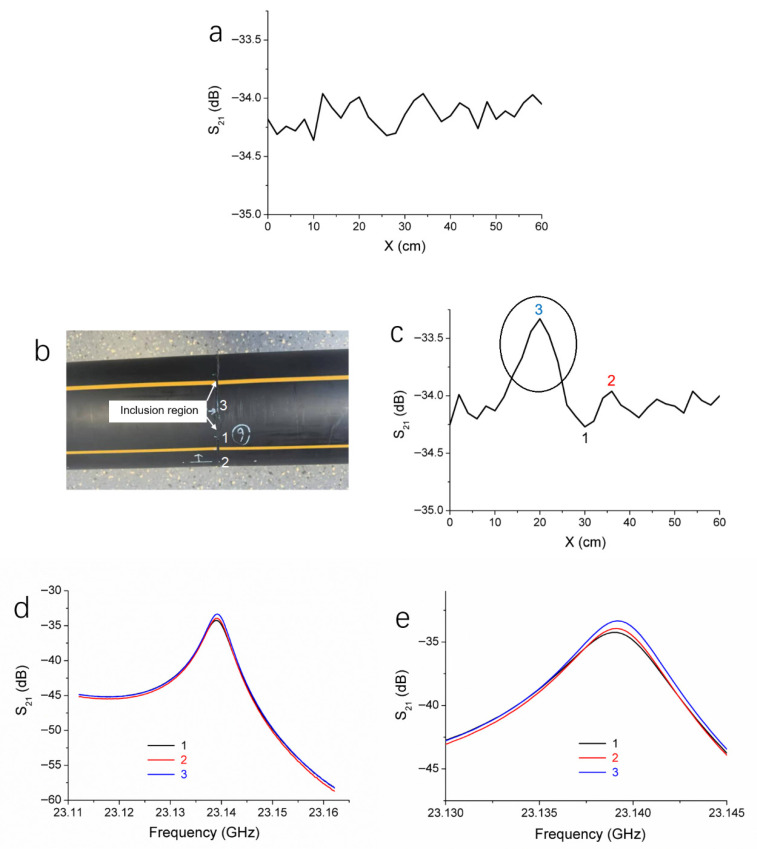
(**a**) Changes of S_21_ magnitude at resonance peak recorded during circumferential scanning for the inclusion-free butt fusion joint. (**b**) A picture of a butt fusion joint exhibiting the talc inclusion region. (**c**) Changes of S_21_ magnitude at resonance peak recorded during circumferential scanning for the butt fusion joint containing the talc inclusion. (**d**) Resonance curves corresponding to the three marked points in (**c**). (**e**) The enlarged view of the resonance curves near the resonance peaks in (**d**).

Talc powder is a common additive used during the manufacturing and welding of HDPE pipes. During the butt fusion of HDPE pipes, the joints may contain talc powder as an inclusion. To investigate the capability of the microwave testing system to detect the presence of talc powder in HDPE pipe butt fusion joints, a fusion joint with talc inclusion and one without inclusion were tested. The microwave resonant probe was positioned at the center of the butt fusion joint, and a 360° scan was performed around the joint’s circumference with a step size of 2 mm. The S_21_ magnitude corresponding to the resonance peak at each point was recorded. The scanning results for the two butt fusion joints are plotted with the circumferential scanning distance on the x-axis and the S_21_ magnitude on the y-axis, as shown in Figure 5. Figure 5a shows the circumferential scanning S_21_ magnitude curve for the joint without talc inclusion. At all circumferential positions, the S_21_ magnitude exhibits small fluctuation. Figure 5b shows the talc inclusion region of the joint, for which the S_21_ magnitude curve obtained by circumferential scanning is shown in Figure 5c. It is clear that in the 15–25 cm region (indicated by the circle), the S_21_ magnitude is noticeably higher, indicating the presence of talc inclusion.

Figure 5d,e display the resonance curves corresponding to three points on the scanning curve in Figure 5c, where point 1 is the highest S_21_ magnitude within the inclusion area, and points 2 and 3 correspond to the highest and lowest S_21_ magnitudes in the defect-free areas, respectively. The S_21_ magnitudes at resonance peaks in the talc inclusion area are higher, suggesting an enhancement of the microwave signal within the resonant cavity. As discussed above, when microwaves propagate from one medium to another, reflections occur at the interface, with the reflected signal’s intensity and phase depending on the relative dielectric constants of the two media. In this study, the relative dielectric constant of the included talc powder is 5.0, while the relative dielectric constant of HDPE is 2.58, showing a significant difference. Microwave reflection at the interface between HDPE and talc powder enhances the reflected signal, altering the resonant state within the cavity and changing the resonance curve. The increase in the S_21_ magnitude indicates that the reflected microwave signal, which is in phase with the incident wave, predominates, resulting in constructive interference within the resonant cavity. Additionally, based on the results in Figure 5d,e, the resonance frequency is almost unaffected by the talc inclusion.

**Figure 6:**
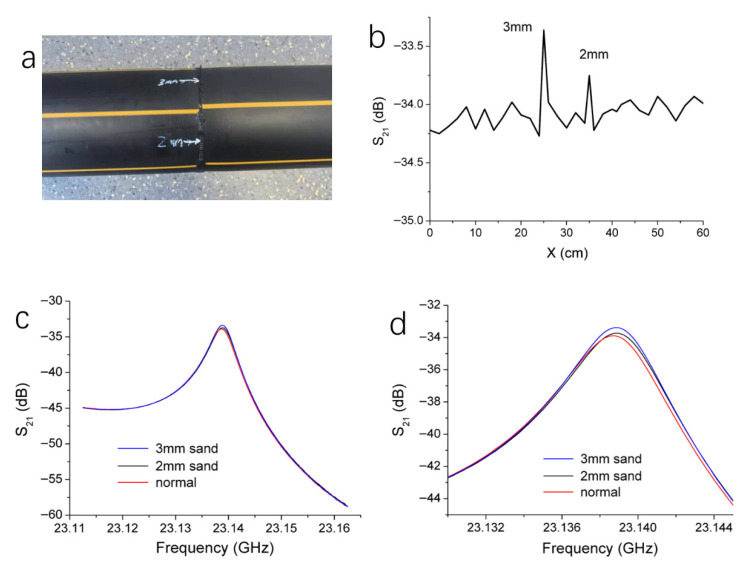
(**a**) A picture of a butt fusion joint containing sand inclusions. (**b**) Changes of S_21_ magnitude at resonance peak recorded during circumferential scanning for the butt fusion joint containing the sand inclusion. (**c**) Resonance curves corresponding to the three marked points in (**b**). (**d**) The enlarged view of the resonance curves near the resonance peaks in (**d**).

In addition to talcum powder, inclusions of sand particles may also occur during the butt fusion welding of HDPE pipe joints. This defect primarily arises from sand particles present on the surfaces of the pipe ends prior to fusion. Herein, a butt fusion joint containing sand inclusions was inspected. Two locations on the joint contained embedded sand particles with diameters of 2 mm and 3 mm, respectively, as shown in Figure 6a.

The probe was aligned with the joint and scanned around the joint surface. The S_21_ magnitude curve versus scanning distance is shown in Figure 6b. At normal locations, the S_21_ magnitude displays small fluctuation. When the probe was moved to the locations containing sand inclusions, noticeable changes in the curve were observed, with the 3 mm sand particle resulting in more pronounced changes. Figure 6c presents the resonance curves at a defect-free location as well as at the positions with the 2 mm and the 3 mm sand particles. Compared to the defect-free region, the S_21_ at the resonance peak increases at the 2 mm inclusion site and further increases at the 3 mm site. Figure 6d provides an enlarged view of the resonance curves near the resonance peak, clearly illustrating the changes induced by the 2 mm and 3 mm sand particles. The S_21_ magnitude increases by 0.23 dB and 0.57 dB, respectively, compared to the defect-free position. The relative dielectric constant of the sand particles is 3.8, smaller than that of the talc powder. The increase in the S_21_ magnitude exhibits a less significant increase compared to the talc powder. This provides a basis for distinguishing between talc inclusion and sand inclusion. Besides, the sand inclusion hardly affects the resonance frequency, similar to the case with talc.

## 4. Conclusions

This study introduces a novel defect detection method based on a microwave resonant probe for evaluating the structural integrity of butt fusion joints in HDPE pipes. The proposed microwave detection system is designed to detect common defects such as cracks, holes, and inclusions within HDPE pipes, with a focus on butt fusion joints. The system works by analyzing variations in resonance frequency and S_21_ magnitude, which change as microwaves interact with defects in the pipe material. Experimental results show that the system is highly sensitive to small defects, including holes of different sizes (ranging from 1 mm to 5 mm in diameter) and surface cracks of varying depths (from 1.5 mm to 4 mm). The method can effectively distinguish defects based on the magnitude reduction in the S_21_ curve and the width of dips in the scanning results. Furthermore, the system demonstrated the ability to identify inclusions such as talc powder and sand particles in fusion joints, revealing significant changes in the resonance curve. These findings suggest that microwave resonant detection offers a robust, non-destructive approach for evaluating HDPE pipe quality, providing accurate and reliable assessments of welding quality and pipeline integrity. This technique has great potential for practical applications in pipeline inspection, particularly in non-metallic materials where traditional methods may be less effective. We will focus on improving the integration level of this microwave nondestructive testing system in the future to enhance its applicability in on-site inspections.

## Data Availability

The original contributions presented in this study are included in the article. Further inquiries can be directed to the corresponding authors.
